# pH-Responsive “Smart” Hydrogel for Controlled Delivery of Silver Nanoparticles to Infected Wounds

**DOI:** 10.3390/antibiotics10010049

**Published:** 2021-01-05

**Authors:** Hanif Haidari, Zlatko Kopecki, Adam T. Sutton, Sanjay Garg, Allison J. Cowin, Krasimir Vasilev

**Affiliations:** 1 UniSA Clinical & Health Sciences, University of South Australia, Adelaide, SA 5000, Australia; Hanif.haidari@mymail.unisa.edu.au (H.H.); Zlatko.kopecki@unisa.edu.au (Z.K.); Sanjay.garg@unisa.edu.au (S.G.); Allison.cowin@unisa.edu.au (A.J.C.); 2 Future Industries Institute, University of South Australia, Mawson Lakes, SA 5095, Australia; adam.sutton@mymail.unisa.edu.au; 3 Academic Unit of STEM, University of South Australia, Mawson Lakes, SA 5095, Australia

**Keywords:** pH-responsive, hydrogel, antibacterial hydrogel, silver nanoparticles, wound infection, controlled delivery, methacrylic acid hydrogel, on-demand release

## Abstract

Persistent wound infections have been a therapeutic challenge for a long time. Current treatment approaches are mostly based on the delivery of antibiotics, but these are not effective for all infections. Here, we report the development of a sensitive pH-responsive hydrogel that can provide controlled, pH-triggered release of silver nanoparticles (AgNPs). This delivery system was designed to sense the environmental pH and trigger the release of AgNPs when the pH changes from acidic to alkaline, as occurs due to the presence of pathogenic bacteria in the wound. Our results show that the prepared hydrogel restricts the release of AgNPs at acidic pH (pH = 4) but substantially amplifies it at alkaline pH (pH = 7.4 and pH = 10). This indicates the potential use of the hydrogel for the on-demand release of Ag+ depending on the environmental pH. *In vitro* antibacterial studies demonstrated effective elimination of both Gram-negative and positive bacteria. Additionally, the effective antibacterial dose of Ag+ showed no toxicity towards mammalian skin cells. Collectively, this pH-responsive hydrogel presents potential as a promising new material for the treatment of infected wounds.

## 1. Introduction

Bacterial wound infections present major therapeutic challenges and have a significant impact on patient health and society [1,2]. Current treatment approaches involve debridement coupled with treatment with antibiotics, which can often result in suboptimal effects, a good example being chronic wounds. Infections are the result of the infiltration of opportunistic bacteria in the wound followed by proliferation, colonization, and biofilm formation [3]. Once a biofilm has been established, it becomes extremely difficult to eradicate. Therefore, eliminating bacterial pathogens from wounds before the infection has developed is of paramount importance. Failure to do this increases the risk of recurrent infections, which can become chronic [4].

The antibacterial properties of silver have been extensively exploited in many healthcare products to protect against infection [5]. Silver nanoparticles (AgNPs) offer broad-spectrum antibacterial activity with a multimodal mechanism of action which helps to reduce the development of antimicrobial resistance [4,6,7]. Despite their significant potential, several challenges remain before AgNPs can be used for routine application in medical practice, including the uncontrolled release of silver ions and potential toxicity to tissue and organs [8,9,10]. Incorporating AgNPs into biocompatible carriers icluding polymeric hydrogel has been demonstrated to be an effective strategy to preserve the properties of the nanoparticles while simultaneously enhancing their biological efficacy [11,12,13,14].

A hydrogel is a three-dimensional network structure of hydrophilic polymers possessing remarkable physical and chemical properties for drug delivery [15,16]. The intrinsic properties of such systems provide excellent swelling capacity, good biocompatibility, and the possibility to fine-tune cargo release characteristics [17]. Particularly, hydrogels can be designed to improve the delivery pathways of AgNPs by acting as the carrier that protects the nanomaterial until it reaches the target site or triggers release driven by pathological stimuli. The stimuli-responsive hydrogel is capable of significant volume change in response to variations in the environment they are exposed to, including pH, temperature, light, and ionic strength [18,19]. pH-sensitive hydrogels containing weak acidic or basic groups within the polymer network are capable of shrinking or swelling at a pH above or below the pKa, caused by ionization of the functional groups in the polymeric network. This results in uptake or loss of water by a hydrogel network, which can be utilized for pH-responsive drug and nanoparticle release [20]. This enables the on-demand delivery of the required amount of AgNPs through responding to a specific endogenous stimulus as a direct result of bacterial infection.

The change in pH which occurs during the development of a wound infection presents an opportunity to develop a responsive carrier for on-demand release of AgNPs. The average pH of healthy skin falls in the range of 4–6; however, the pH is elevated in the presence of bacterial colonization of a wound [21]. Depending on the extent of the infection, the pH becomes alkaline, reaching 7.3–9.8 [22]. A “smart” hydrogel system that is capable of selectively releasing embedded AgNPs in response to wound pH changes would present a major advance in the delivery of AgNPs. The infiltration of the wound by a pathogen would trigger a burst of AgNPs to rapidly control infection and prevent further colonization, while in the absence of pathogenic bacteria, the hydrogel system would produce minimal therapeutic release. Indeed, the usefulness of such responsive systems has been reflected by several recent studies for the controlled delivery of antibacterial therapeutics for topical wound applications including antibiotics [23,24] and antimicrobial peptides [25,26].

**Scheme 1:**
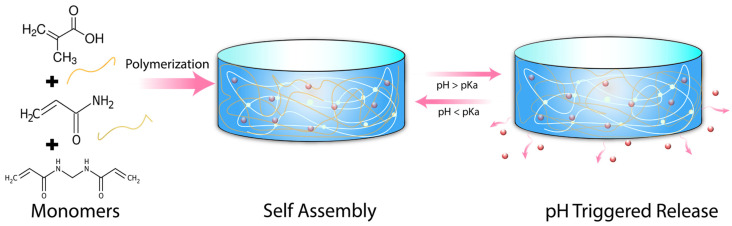
Schematic illustration of methacrylic acid hydrogel synthesis with pH triggering the release of therapeutics.

In this work, we have developed a facile synthetic procedure to polymerize methacrylic acid (mAA) in combination with acrylamide (Amm) crosslinked with N, N’-Methylenebisacrylamide (MBMa) to establish a sensitive pH-responsive hydrogel delivery system for AgNPs, Scheme 1. The presence of an ionizable, anionic acid group from mAA allows phase transition to occur above ~pH 5, owing to the deprotonation of the carboxyl group [27]. The phase transition results in a more hydrophilic polymer network, leading to greater water uptake and increased release of AgNPs.

We demonstrate that the prepared hydrogel possesses relevant swelling capacity in response to changes to the environmental pH. We also show that the AgNPs loaded hydrogel can sense the local pH and modulate the therapeutic release of AgNPs according to the pathological conditions where the release is significantly amplified in the alkaline range. The antibacterial efficacy of the system was evaluated against both Gram-negative and positive bacteria, showing a strong inhibitory effect. Importantly, we also demonstrate that the AgNPs loaded hydrogel has no toxicity to human foreskin fibroblast cells. Therefore, successful and controlled loading of AgNPs into pH-responsive hydrogel could provide benefits for selective and efficacious treatment of wound infection compared to conventional therapies.

## 2. Results and Discussion

### 2.1. Synthesis of AgNPs@MSA

**Figure 1:**
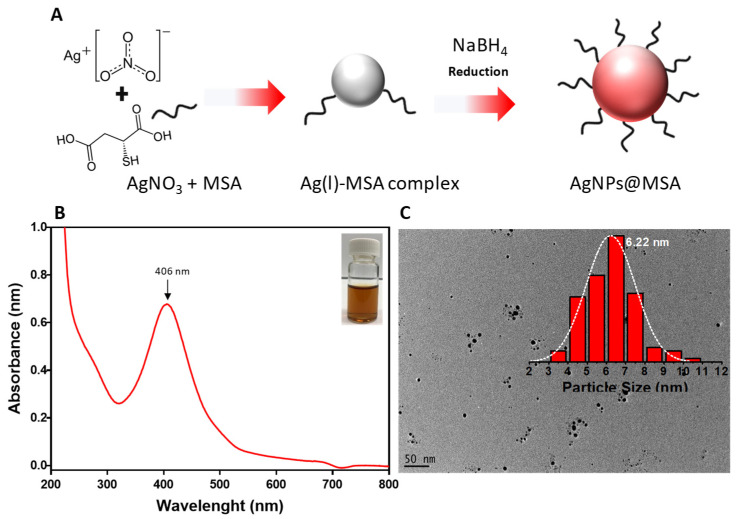
(**A**) Schematic illustration of AgNPs formation via chemical reduction. (**B**) UV–vis absorption spectra of AgNPs; the inset is a photograph of the AgNPs@MSA solution. (**C**) TEM image of the AgNPs; the inset shows the nanoparticle size distribution.

Mercaptossucinic acid (MSA) protected AgNPs were synthesized via facile one-pot chemical reduction using sodium borohydride as a reducing agent as schematically depicted in Figure 1A. The colorless solution of the Ag-thiol complex turned dark brown upon addition of NaBH_4_, Figure 1B (inset). First, the synthesized AgNPs were characterized by UV-Vis spectroscopy. The spectrum shows a clear surface plasmon band with a maximum of ~406 nm indicating the successful formation of AgNPs, Figure 1B. The narrow absorption peak suggests colloidal stability and the absence of aggregation [28]. The morphology of the AgNPs was visualized by transmission electron microscopy (TEM), and the particle size was determined from the images in Figure 1C. The AgNPs were spherical and had a relatively narrow size distribution. The average core size of the AgNPs was 6.2 nm, and their size distribution could be fitted with a Gaussian curve.

### 2.2. Preparation of pH-Responsive Hydrogel

The poly(mAA-co-AAm) hydrogel was synthesized using free radical polymerization similar to reported procedures but with significant optimization [22]. These changes included reducing the material mechanical strength to provide a more spreadable product useful for topical applications and improving pH-responsive properties. The synthetic process was optimized for simple and rapid polymerization without requiring heating or degassing to produce a robust clinically relevant hydrogel. Briefly, the optimized ratio of mAA (100 µL) and AAm (240 mg/mL) was crosslinked by MBMa (4 mg/mL), while the TEMED (100 µL) and APS (80 mg/mL) served as a free radical stabilizer to promote hydrogel polymerization. The optimized hydrogel constituents and ratios can be found in the method section. Following complete polymerization, the hydrogel underwent successive washing to completely remove residual monomers. The purification was verified using liquid chromatography. Purification of the hydrogel did not lead to any noticeable changes in the physical properties of the material. In the last step, AgNPs were encapsulated by physical adsorption to obtain a homogenous distribution across the hydrogel network. The absorption of AgNPs within the hydrogel network was observed, where a transparent hydrogel became brownish in color after 24 h of loading.

### 2.3. pH-Dependent Swelling and Rheological Properties of the Hydrogel

**Figure 2:**
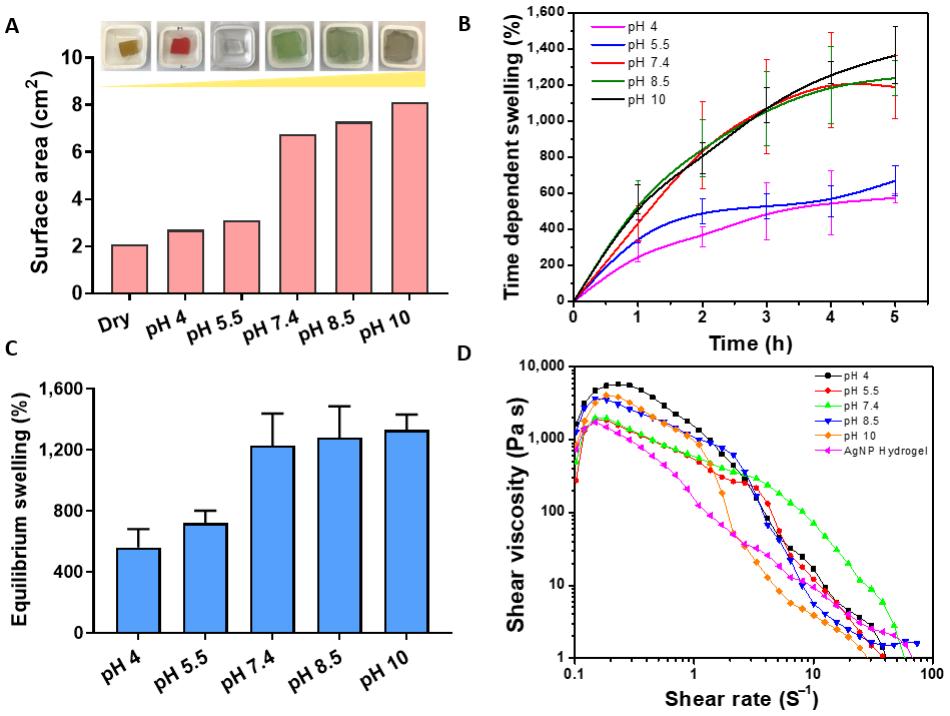
(**A**) Change in surface area (cm^2^) of the hydrogel in response to different pH (4–10) with the corresponding hydrogel photo after equilibrium swelling. The colors are due to the commercial pH buffer used. (**B**) Time dependence swelling kinetics after 5 h of swelling. (**C**) The difference in hydrogel swelling rate after reaching an equilibrium state. (**D**) Measurements of hydrogel mechanical properties measured by shear rate after equilibrium state.

The swelling of the hydrogel was first analyzed by determining changes in surface area over time, followed by the gravimetric method to determine the weight change. A known weight of dried hydrogel was immersed in PBS solution having pH ranging from 4 to 10. In this case, pH 4 and pH 5.5 represent healthy skin, pH 7.4 (acute wound infection) while pH 8.5 & 10 presents the environment of typical chronic wound infection. At predetermined time intervals, hydrogels were removed, the residual liquid from the surface was blotted and the weight was recorded. We hypothesized that at high pH, the carboxyl group of the methacrylic acid would be deprotonated and negatively charged, thereby generating electrostatic repulsion between molecular chains, resulting in a significant expansion of the hydrogel network [29]. In contrast, at lower pH, the carboxyl group would be protonated, resulting in a collapse of the hydrogel network and shrinkage. Indeed, as shown in Figure 2A, there was a dramatic change in hydrogel volume as measured by the change in surface area in response to pH. The surface of the hydrogel incubated at pH 4 and 5.5 remained very similar to the original state (dry 2 cm^2^), whereas the increase in pH to 7.4, 8.5, and 10 resulted in significant swelling to 7 cm^2^, 7.2 cm^2^, and 8 cm^2^, respectively Figure 2A. This is consistent with a previous study showing changes in methacrylic acid hydrogel capsules in response to pH solution from pH ~5 to alkaline range (7–8) [30]. The digital photographs in Figure 2A show the surface area expansion of the hydrogel after attaining maximum swelling capacity.

The swelling kinetics was also measured based on the weight change over time. As shown in Figure 2B, the swelling index significantly increased as the pH of the swelling medium increased, where the hydrogel in the pH range of 7.4–10 showing the highest rate of swelling. The maximum swelling at 5 h is plotted in Figure 2C, and follows a consistent trend, as the swelling is determined via measuring the surface area Figure 2A. The data indicates that the swelling of the hydrogel, in response to environmental pH, had a substantial effect on its physical properties [31]. We expected that the observed swelling behavior would influence the diffusion of AgNPs out of the hydrogel in response to environmental pH and provide a responsive controlled release system. Furthermore, the rheological behavior of the hydrogel was also assessed to determine its viscoelastic behavior after equilibrium swelling. As shown in Figure 2D, the viscosity of the hydrogels decreased as the shear rate increased, demonstrating the typical shear thinning behavior [32]. Based on this, the high crosslinking and dense structure of this hydrogel restricts the repulsive force between chains and improves the physical properties greatly withstanding external force.

### 2.4. Hydrogel Morphology

**Figure 3:**
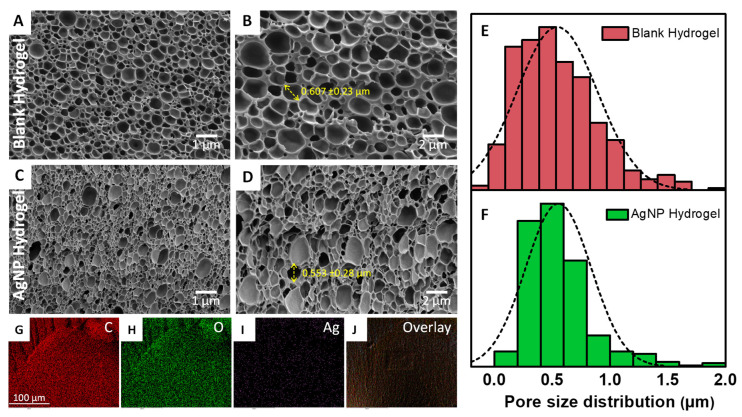
(**A**–**D**) SEM micrographs of both blank and AgNP hydrogel at two different magnification 10K X (left panel) and 20K X (right panel). The yellow arrow shows the representative pore size. (**E**,**F**) Hydrogel size distribution of both blank and AgNP-loaded hydrogel. (**G**–**J**) EDS elemental mapping of carbon (C), oxygen (O), silver (Ag), and overlay images.

The morphology of both the blank and AgNP-loaded hydrogels was assessed by scanning electron microscopy (SEM). For this study, the fully swollen hydrogels were dehydrated with ascending ethanol concentration and completely dried with hexamethyldisilazane (HMDS). The SEM micrographs exhibited highly porous structures, Figure 3A–D. It appears that after loading with AgNPs the pore size slightly decreases as reflected in the pore size distribution analysis presented in Figure 3E,F. The average pore size of the blank hydrogel was 0.607 ± 0.23 µm while this parameter for the AgNP hydrogel was 0.553 ± 0.28 µm. This indicates that the AgNP encapsulation in the hydrogel network had only a small effect on the pore size of the hydrogel. The AgNPs could not be visualized by SEM due to their very small size (~6 nm). To confirm the incorporation of AgNPs in the hydrogel network, we used energy dispersive spectroscopy (EDS) elemental mapping. The major elements present in the hydrogel are shown in Figure 3G–J. The data indicated that AgNPs were homogenously deposited across the hydrogel network (see Figure 3I) and visible in the overlay images (see Figure 3J).

### 2.5. pH-Dependent Release Kinetics

**Figure 4:**
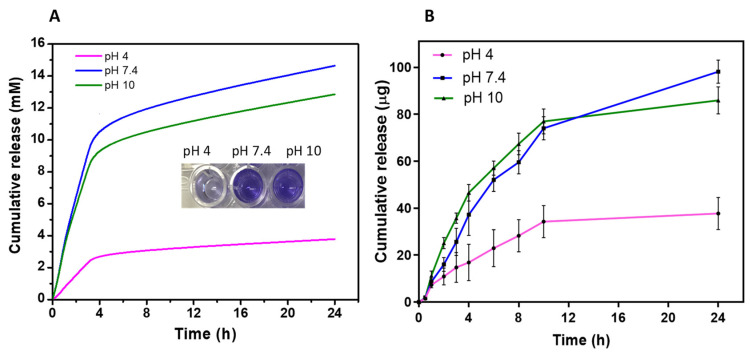
(**A**) pH-dependent cumulative release of crystal violet dye from the hydrogel in response to different pH within 24 h. The inset showing representative digital photographs of crystal violet eluted from the gel. (**B**) Cumulative release of AgNPs from the hydrogel at low, medium, and high pH within 24 h. Data are shown as mean ± SD.

Thus far, the hydrogel showed a well-defined switching behavior in response to environmental pH. To gain insight into its on-demand release capacity, the hydrogel was loaded with crystal violet that was released at low (pH = 4), medium (pH = 7.4), and high (pH = 10) pH, as measured over a 24 h period. As shown in Figure 4A, the amount of crystal violet released from the hydrogel was highly dependent on the pH of the solutions. The hydrogel immersed in pH 4 showed a significantly slower release rate compared to hydrogels exposed to pH 7.4 or 10. This is also reflected in representative digital images showing substantial differences in color intensity of the released crystal violet (see Figure 4A (inset)). Consistent with the swelling studies (Figure 2), the hydrogel demonstrated satisfactory pH sensitivity in response to change in pH solution. This occurred due to the greater expansion of the hydrogel network at higher pH, allowing faster diffusion of water and compounds in and out of the material.

After the proof of concept using crystal violet, we then examined the amount of AgNPs released from the hydrogel over time. The concentration of AgNPs in solution prior to hydrogel loading was quantified as (110 µg/mL). The cumulative amount of AgNPs released within 24 h is shown in Figure 4B. Consistently, the hydrogel showed significantly slower AgNPs release at pH 4 compared to pH 7.4, and 10 which shows substantial release capacity. The release profile of the AgNP-loaded hydrogel at pH 7.4 and 10 showed an initial fast release in the first 6 h followed by a steady release for the remainder of the time interval studied. At 24 h, the hydrogel released 98 µg of AgNPs at alkaline pH compared to 37 µg in acidic pH. This was expected, as the hydrogel structure transforms into a collapsed state at acidic pH and restricts the movement of AgNPs within the polymer networks. In contrast, hydrogel exposed to alkaline pH was in a strong hydrated state, resulting in easier diffusion of the AgNPs out of the material. These results demonstrated that the hydrogel possesses the desired properties of the pH-dependent release of AgNPs. In the context of wound treatment, the hydrogel would release a relatively small amount of AgNPs in the absence of bacteria. However, if pathogens are colonizing the wound, the pH of the environment would increase, leading to a responsive delivery of AgNPs to eradicate the bacteria.

### 2.6. Antibacterial Activity

**Figure 5:**
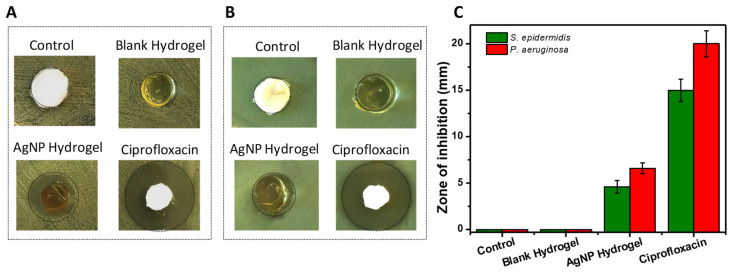
(**A**,**B**) Representative images of the zone of inhibition of negative control, blank hydrogel, AgNPs loaded hydrogel, and Ciprofloxacin as positive control tested against *S. epidermidis* (**A**) and *P. aeruginosa* (**B**). (**C**) Quantification of the corresponding zones of inhibition. Data are shown as mean ±SD.

The antibacterial activity of the AgNP-loaded hydrogels was examined against Gram-negative and -positive bacteria, *P. aeruginosa*, and *S. epidermidis,* respectively. These bacteria are representative of common pathogens found in infected wounds and present a clinical challenge [33]. The results of the zone of inhibition assay carried out using PBS as the negative control, blank hydrogel, AgNP-loaded hydrogel, and Ciprofloxacin as positive control is shown in Figure 5A–C. The AgNP-loaded hydrogels generated a clear zone of growth inhibition compared to the blank hydrogels where such zones were not observable after 18 h. The mean zone of inhibition measured was 5 mm and 6.5 mm for *S. epidermidis* and *P. aeruginosa*, respectively. The results indicate that the blank hydrogels did not contribute to the antibacterial properties of the material. Importantly, the results also demonstrated that AgNPs can be released from the hydrogels at a sufficient rate to eliminate the bacteria.

**Figure 6:**
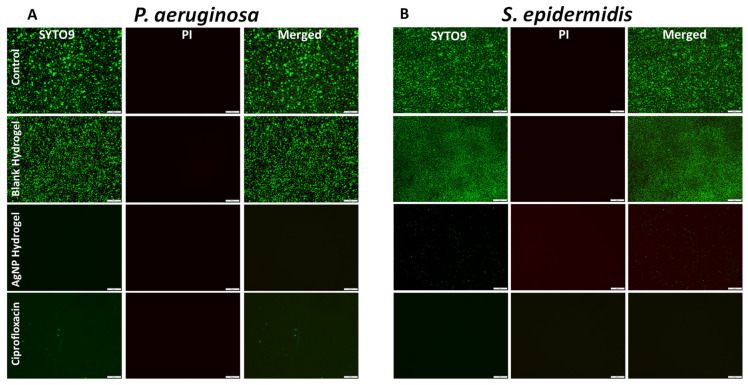
(**A**,**B**) Representative fluorescence microscopy images of *P. aeruginosa* (left panel) and *S. epidermidis* (right panel) stained with Live/Dead viability kit after 24 h of incubation with control, blank hydrogel, AgNPs loaded hydrogel, and Ciprofloxacin. Scale bar 50 µm.

Next, we studied the bacterial viability using the so-called Live/Dead assay. The kit contains two dyes: SYTO9 which stains all cells and emits green fluorescence while PI stains cells with compromised membranes (emitting red fluorescence). Representative fluorescent microscopy images are shown in Figure 6A,B. A healthy population of green-stained, healthy bacterial cells can be seen in the wells where control or blank hydrogels were inoculated. No dead cells were observed with the blank hydrogel, which further confirmed that the blank hydrogel itself is nontoxic to bacterial cells. In response to AgNP-loaded hydrogels or antibiotics, no viable cells were detected. This indicates that the bacterial cells were not able to colonize the surface of the wells, since planktonic cells were killed and debris was washed away during the staining process, as observed and reported previously [34]. The data so far demonstrate that the AgNP-loaded hydrogel is very effective in eliminating both Gram-positive and negative bacteria.

### 2.7. In Vitro Cytotoxicity Evaluation

**Figure 7:**
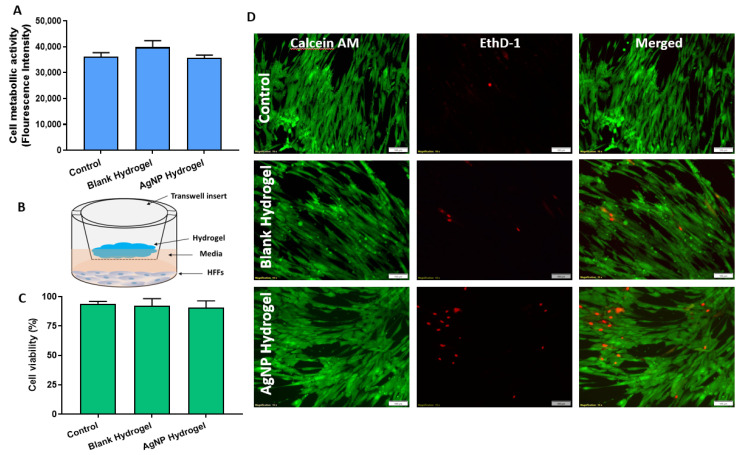
(**A**) Metabolic activity of human fibroblasts in the presence of blank and AgNP-loaded hydrogels as measured by the resazurin assay. (**B**) Schematic illustration of the indirect test of cell viability by transwell assay. (**C**) Corresponding statistical analysis of cell viability based on fluorescence microscopy images. (**D**) Representative fluorescence microscopy images of HFFs stained with calcein AM (green) and EthD-1 (red) to determine the ratio of Live/Dead cell. Data are shown as mean ±SD, scale bar 100 µm.

In addition to antibacterial efficacy, a hydrogel used for wound treatments should also have good biocompatibility and possess mechanical properties that support host cell interactions that are beneficial for the wound healing process [35]. In this experiment, potential cytotoxicity caused by the AgNP hydrogels was first assessed using resazurin which measures the metabolic activity of the cells. AgNP-loaded and blank hydrogels were incubated with representative skin cells using a human foreskin fibroblast cell line (HFFs). Fibroblasts are one of the most important cell types directly involved in the wound healing process [36]. As shown in Figure 7A, the antibacterial hydrogel showed no toxic effect to HFFs, and was very similar to untreated controls. The HFFs maintained their maximum metabolic activity in the presence of both the blank and AgNP hydrogel, which indicated that the material was biocompatible.

The biocompatibility of the hydrogels was further confirmed using the Live/Dead viability assay. The setup for this experiment is schematically presented in Figure 7B. Briefly, a known quantity of hydrogel (1 g) was placed in a transwell insert while submerged on top of a cell monolayer containing the culture media. This approach avoids the direct contact of the material with the cell monolayer while allowing any leachables from the hydrogel to interact with the cell and represents a simulation of typical wound dressing applications [37]. After 24 h of exposure, the cell monolayer was stained with a Live/Dead mammalian cell viability kit. Herein, the viable cells are stained with intense green fluorescence (calcein-AM) while the damaged cells are stained with red (EthD-1). Representative fluorescent micrographs and quantification are presented in Figure 7C,D. The fluorescent staining shows a dense HFFs monolayer with intact morphology which was able to maintain the cell layer integrity comparable to control. The quantitative ratio of live and dead cells indicates >90% cell viability compared to control Figure 7C. The results are in agreement with the metabolic assay (shown in Figure 7A) demonstrating excellent biocompatibility of the AgNP-loaded hydrogel, compared to untreated control.

Taken together, this study has demonstrated the possibility to integrate AgNPs into a biocompatible, pH-responsive hydrogel capable of eliminating bacterial pathogens without inducing negative effects on mammalian cells. Future studies are required to demonstrate the utility of this newly-designed material in in vivo animal models. Nevertheless, this platform presents a promising strategy for the incorporation and release of on-demand AgNPs into a pathological environment to eliminate invading bacteria without inducing adverse effects on host cells and tissue.

## 3. Materials and Methods

### 3.1. Materials

AgNO_3_ (99%), mercaptossucinic acid (MSA), sodium borohydride (NaBH_4_), Methacrylic acid (mAA, 99%), Ammonium persulfate (APS, 98%), Acrylamide (AAm, 99%), Tetramethylethylenediamine (TEMED, 99%), nitric acid (HNO_3_), sodium hydroxide (NaOH), Hexamethyldisilazane (HMDS) and N, N’-Methylenebisacrylamide (MBAm, 99%) were all purchased from Sigma Aldrich, (Sydney, Australia). All other reagents used throughout the study were analytical grade, commercially available, and used without further purifications. Ultrapure water with 18.2 MΩ (Millipore) was used in all synthesis and preparations.

### 3.2. Synthesis of AgNPs

Mercaptosuccinic acid (MSA) -coated AgNPs were prepared following our previously established procedure, with slight modification [28]. Briefly, 6 mL of 2 mM AgNO_3_ was mixed with 2.5 mL of 2 mM MSA under ice-cold condition. Then, 0.25 mL of 0.05 M NaBH_4_ was added to the mixture in a drop-wise manner under vigorous stirring. The solution color changed from pale to dark brown immediately and the reaction was continued for another 24 h at 500 rpm. The slow progress of color change from pale to dark brown confirmed the formation of AgNPs@MSA. After synthesis, the NP solution was purified through dialysis (Pur-A-Lyzer™ Maxi Dialysis Kit; MWCO 3.5 kDa, Perth, Australia) against ultrapure water, lyophilized using (Modulyo freeze, Asheville, USA), and stored 4 °C for further use. The concentration of AgNPs was determined using inductively coupled plasma optical emission spectrometry (ICP-OES) similar to our previous study [28].

### 3.3. Characterization of AgNPs

The optical properties of the AgNP solution were characterized using UV-Vis spectroscopy (Shimadzu 2600, Japan). The primary size of the AgNPs was determined by Transmission Electron Microscopy (TEM) (JEOL JEM-2100F-HR, Tokyo, Japan) operating at 200 kV. The AgNPs size distribution was determined by counting over 200 nanoparticles using ImageJ software (Fiji, USA) 1.53a.

### 3.4. Preparation of pH-Responsive Hydrogel

The pH-responsive hydrogel was prepared using a free radical polymerization approach adapted from a previous procedure [22]. The procedure was adapted to obtain a product more suitable for topical administration to infected wounds that were also more sensitive to pH changes. The typical synthesis was as follows, 1 mL of methacrylic acid (mAA), 1.67 g of acrylamide (AAm), 500 µL of tetramethylethylenediamine (TEMED), 16.35 mg of N, N’-methylenebisacrylamide (MBAm), and 5.5 mL of MilliQ water were combined in a Schott bottle. The solution was sonicated for 5 min to dissolve all the components then poured into a plastic petri dish. Then 1.18 mL of ammonium persulfate (APS) dissolved in water at 80 mg/mL was added to initiate the polymerization at room temperature (~20 °C). The petri dish was swirled to mix the two solutions. The reaction was left overnight with a hydrogel visibly forming after 1 h. The final poly(mAA-co-AAm) based hydrogel was purified by 50 L ultrapure water being flowed through the swollen hydrogel for 12 h continuously, completely removing residual monomers as confirmed by liquid chromatography. Subsequently, the purified hydrogel was cut into 1 cm^3^ cube size and dried in a vacuum oven at 45 °C, overnight for 18 h. The AgNPs were loaded into the hydrogel by immersing a dried piece of a hydrogel in 5 mL of the AgNPs suspension overnight at ambient temperature. Swollen AgNPs containing hydrogels were washed with 3 × 5 mL of water by fully submerging the hydrogel in water to remove any remaining surface bound AgNPs.

### 3.5. Scanning Electron Microscopy (SEM)

The microstructure and pore size of the AgNP hydrogels or blank controls were examined using scanning electron microscopy (SEM) (Carl Zeiss Crossbeam 540, Oberkochen, Germany). The hydrogel samples were fully swollen in MilliQ water and then subjected to ethanol dehydration using a series of ethanol solutions in ascending concentration (20%, 50%%, 70%, 80%, 90%, and 100%) for 15 min at each concentration. Samples were then chemically dried using hexamethyldisilazane (HMDS) in a series of (1:2 ethanol, and finally 2×100% HDMS) for 20 min each. Hydrogel samples were left to dry in the airflow cabinet followed by overnight storage in a desiccator. The dried samples were cracked by a razor and mounted on aluminium stubs, sputter-coated with 2 nm of platinum, and imaged using SEM at 2 kV. The composition of major elements present on a surface of AgNP-loaded hydrogels was analyzed using energy dispersive X-ray spectrometer (EDS) mapping at an acceleration voltage of 15 kV (SEM Carl Zeiss Crossbeam 540 with SDD EDS, Oberkochen, Germany). The pore size of the hydrogel was determined by measuring the pores from SEM images using ImageJ software (Fiji, USA) 1.53a.

### 3.6. pH-Responsive Swelling

The swelling ratio of the hydrogel at different pH solutions was assessed based on the gravimetric method following established protocols [38]. Preweighed, dry hydrogels (approx. 0. 3 g) were immersed in different phosphate buffer saline (PBS) 0.1 M at different pHs (4, 5.5, 7.5, 8.5, and 10) representative of different physiological pH levels of normal skin and infected wounds. The hydrogel swelling capacity was measured until the equilibrium state was reached. The pH of the PBS solution was adjusted by 1 M HCI and NaOH solution using a pH meter. The hydrogel sample was withdrawn from the buffer at different time intervals and was gently blotted with filter paper to remove surface water and immediately weighed. The swelling percentage (SP) of the hydrogel was calculated from the weight of the sample at equilibrium swelling (We) and the weight of the dried sample (Wd) SP% ((We-Wd))/Wd × 100.

### 3.7. Rheological Characterization

The rheological experiments were carried out on a rheometer (Malvern Kinexus TA instrument, Selb, Germany) using a parallel plate with a diameter of 25 mm. A sample of both blank and AgNP-loaded hydrogel was measured. The oscillatory shear rate sweep was carried out at an ambient temperature and a shear rate of 1–100 s^−1^ to determine the viscoelastic behavior.

### 3.8. pH-Responsive Analysis of Hydrogel Loaded with Crystal Violet

To examine pH-dependent disassembly and release, dried hydrogel samples were immersed in crystal violet solution until equilibrium swelling. The fully swollen hydrogels were removed from the solution and gently washed with MilliQ to remove excess crystal violet from the surface. Approx. 2 g of hydrogel loaded crystal violets was subjected to pH-dependent release using PBS solution 0.1 M at different pH (4, 7.5, and 10) in 10 mL buffer solutions. The system was maintained at 37 °C ± 0.5 °C in a thermostatically controlled shaking incubator at 90 rpm. Aliquots of 200 µL were withdrawn at time intervals of 0.5, 1, 2, 3, 4, 6, 8, and 24 h which were replaced by the same amount of fresh PBS at appropriate pH solution. The fluorescence intensity of the released dye was measured using a plate reader (BMG LabTech, Melbourne, Australia), and the cumulative amount calculated using the known standard concentrations.

### 3.9. pH-Dependent Release of AgNP- Loaded Hydrogel

The in vitro release of AgNPs from the prepared hydrogel was studied in a similar approach as described above. AgNP-loaded hydrogel samples of (2 g) were immersed into a 50 mL falcon tube (receptor compartment) containing 10 mL of PBS at different pH solutions (4, 7.5, and 10) as the release medium. The system was maintained at 37 °C ± 0.5 °C in a thermostatically controlled shaking incubator at ~100 rpm. Aliquots of 2 mL were withdrawn at intervals of 0.5, 1, 2, 3, 4, 6, 8, 10, and 24 h which were replaced by the fresh solution (2 mL) to maintain a constant volume. The concentration of the AgNPs was analyzed by a calibration curve using UV-Vis spectroscopy (Shimadzu, Sydney, Australia). The experiment was conducted using three replicates on two different occasions.

### 3.10. In Vitro Antibacterial Assessment

#### Bacterial Culture

*Staphylococcus epidermidis* ATCC 35984 and *Pseudomonas aeruginosa* PAO1 were streaked on Tryptic Soy Agar (TSA) plates. Overnight bacterial cultures were prepared by inoculating a single colony into 10 mL Tryptic Soy Broth (TSB) medium incubated overnight (18 h) at 37 °C in a shaking incubator. The optical density was measured at 660 nm (OD660) and standardized by diluting the overnight culture to OD 0.25 which equates to 2.5 × 10^8^ CFU/mL. The resulting standardized inoculum was further diluted as specified below.

### 3.11. Zone of Inhibition

Overnight culture of bacteria was adjusted to OD 660nm 0.25 and diluted further to obtain 1 × 10^6^ CFU/mL). The bacterial lawn was prepared by adding 100 µL of diluted inoculum onto the TSA agar surface (Oxoid, Thermo Fisher Scientific, Sydney, Australia) and spread uniformly using a disposable spreader. Each of the test samples (AgNP hydrogel and blank hydrogel) was uniformly cut into circular discs of 10 mm in diameter using a punch biopsy and sterilized under UV lamps for 30 min. Ciprofloxacin antibiotic (100 µL) was added onto a filter paper disc at a final concentration of 25 µg/mL serving as the positive control. The agar plates were incubated at 37 °C overnight (18 h) and the zone of inhibition (mm) was measured the following day.

### 3.12. Live/Dead Bacterial Viability Assay

The bacterial viability was measured using the Live/Dead Baclight™ viability kit as per the manufacturer’s manual (Invitrogen, Thermo Fisher Scientific, Sydney, Australia). An overnight culture of bacteria (*S. epidermidis* and *P. aeruginosa*) was adjusted to 1 × 10^6^ CFU/mL in a TSB and was added in a 12 well plate containing a sterile glass coverslip. Then, transwell inserts with test samples were submerged in the wells containing 2 mL of culture broth, and an additional 1 mL of broth was added to the inserts. After overnight incubation at 37 °C, the wells were gently washed with PBS to remove unadhered bacteria. Then the bacterial cells were stained with a working concentration (1:1) of 3 µL/mL of (SYTO9 and Propidium Iodide) in PBS. The samples were incubated in the dark for 15 min at 25 °C, followed by a PBS wash. The glass coverslips were mounted on a microscope slide to be imaged via the Olympus IX81 epifluorescence microscope (Olympus, Tokyo, Japan).

### 3.13. In vitro Cytotoxicity Assay

Cell viability was determined using the resazurin and Live/Dead assay. The human foreskin fibroblasts (HFFs) were seeded in 96 and 12 well tissue culture plates at a density of 4 × 10^4^ cells/well and 1 × 10^5^ in DMEM, supplemented with 10% FBS and 5% penicillin and streptomycin and incubated at 37 °C in 5% CO_2_ for 24 h. Extracts of the samples were prepared by incubating the AgNP hydrogel in DMEM (1:1) for 24 h at 37 °C incubator. After reaching 90% confluency, the wells were washed with PBS, and subsequently, cells were treated with hydrogel extracts at 100 µL. After further incubation of 24 h, the cells were washed and resazurin dye was added. The stock concentration of resazurin (110 µg/mL) was diluted in 1:10 in DMEM and the working solution was added to the wells. The cells were incubated for 2 h at 37 °C incubator and then the fluorescence intensity of the dye was measured using a plate reader (BMG LabTech, Melbourne, Australia).

Cells grown on 12 well tissue culture plates were used for Live/Dead staining. Briefly, hydrogel samples were UV sterilized and then placed in a transwell insert to be applied on top of the cell monolayer without direct contact of the material to the cell. Each well contained 3 mL of DMEM as a culture medium. The treatment groups were incubated at 37 °C in 5% CO_2_ for 24 h before cell staining with Live/Dead mammalian cell staining (Invitrogen, Thermo Fisher Scientific, Sydney, Australia). A stock solution of 4 mM calcein AM and 2 mM ethidium homodimer-1 (EthD-1) was prepared according to the manufacturer’s instruction (Molecular Probes, 03224, Thermo Fisher Scientific, Sydney, Australia). Thereafter, a working concentration of (2 µM calcein and 4 µM EthD-1) was then added directly to the cells in 200 µL per sample. After incubation for 15 min in the dark, samples were briefly washed and imaged via Olympus IX81 epifluorescence microscope (Olympus, Tokyo, Japan).

## 4. Conclusions

In summary, we report the preparation and evaluation of a pH-responsive hydrogel integrating silver nanoparticles as an active component for the potential treatment of wound infection. The hydrogel was able to rapidly switch from a collapsed state in a lightly acidic environment (normal wound) to a hydrated state in an alkaline environment (infected wound). This pH-dependent expansion triggered a significantly greater rate of AgNP release at alkaline pH (7.4 and 10) compared to that in acidic (pH 4). The antibacterial efficacy of the AgNP-loaded hydrogel was verified against both Gram-negative and -positive bacteria. Importantly, the AgNP-loaded hydrogel showed no toxicity to mammalian cells, suggesting a high degree of biocompatibility. The results presented in this paper confirm the synthesis of a novel, AgNP-integrated, pH-responsive hydrogel that is capable of on-demand release of AgNPs if pathogenic bacteria are present in a wound. Such an approach may provide a more efficient way forward for the clinical management of chronically infected wounds.

## Data Availability

The data presented in this study are available on request from the corresponding author.
